# Supplementary material for: Pyruvate Dehydrogenase Kinase 1 inhibition mediated oxidative phosphorylation enhancement in cartilage promotes osteoarthritis progression
Source: BMC Musculoskelet Disord. 2023 Jul 20;24:597. doi: 10.1186/s12891-023-06585-6 (PMC10357736; doi:10.1186/s12891-023-06585-6)
Supplement: Supplementary file 1 — Additional file 1. [file 12891_2023_6585_MOESM1_ESM.zip › MF.Gsea.1653627316242/GOMF_TRANSCRIPTION_COREGULATOR_ACTIVITY.html]

Details for gene set GOMF\_TRANSCRIPTION\_COREGULATOR\_ACTIVITY[GSEA]

|  || Dataset | 55457\_collapsed\_to\_symbols.cls.cls #OA\_versus\_normal.cls.cls #OA\_versus\_normal\_repos |
| Phenotype | cls.cls#OA\_versus\_normal\_repos |
| Upregulated in class | normal |
| GeneSet | GOMF\_TRANSCRIPTION\_COREGULATOR\_ACTIVITY |
| Enrichment Score (ES) | -0.27874327 |
| Normalized Enrichment Score (NES) | -1.4355103 |
| Nominal p-value | 0.0 |
| FDR q-value | 0.23921075 |
| FWER p-Value | 1.0 |
Table: GSEA Results Summary

  

Fig 1: Enrichment plot: GOMF\_TRANSCRIPTION\_COREGULATOR\_ACTIVITY      
 Profile of the Running ES Score & Positions of GeneSet Members on the Rank Ordered List

  

| SYMBOL | TITLE | RANK IN GENE LIST | RANK METRIC SCORE | RUNNING ES | CORE ENRICHMENT || 1 | MED7 | mediator complex subunit 7 [Source:HGNC Symbol;Acc:HGNC:2378] | 83 | 0.939 | 0.0008 | No |
| 2 | RNF14 | ring finger protein 14 [Source:HGNC Symbol;Acc:HGNC:10058] | 90 | 0.926 | 0.0076 | No |
| 3 | ARL2BP | ADP ribosylation factor like GTPase 2 binding protein [Source:HGNC Symbol;Acc:HGNC:17146] | 208 | 0.813 | 0.0046 | No |
| 4 | TSG101 | tumor susceptibility 101 [Source:HGNC Symbol;Acc:HGNC:15971] | 284 | 0.763 | 0.0046 | No |
| 5 | TRIM14 | tripartite motif containing 14 [Source:HGNC Symbol;Acc:HGNC:16283] | 343 | 0.726 | 0.0057 | No |
| 6 | TLE6 | "TLE family member 6, subcortical maternal complex member [Source:HGNC Symbol;Acc:HGNC:30788]" | 385 | 0.711 | 0.0080 | No |
| 7 | SLC30A9 | solute carrier family 30 member 9 [Source:HGNC Symbol;Acc:HGNC:1329] | 390 | 0.710 | 0.0133 | No |
| 8 | RAP2C | "RAP2C, member of RAS oncogene family [Source:HGNC Symbol;Acc:HGNC:21165]" | 404 | 0.705 | 0.0178 | No |
| 9 | DNMT3A | DNA methyltransferase 3 alpha [Source:HGNC Symbol;Acc:HGNC:2978] | 406 | 0.704 | 0.0233 | No |
| 10 | EID1 | EP300 interacting inhibitor of differentiation 1 [Source:HGNC Symbol;Acc:HGNC:1191] | 456 | 0.679 | 0.0247 | No |
| 11 | DHRS7B | dehydrogenase/reductase 7B [Source:HGNC Symbol;Acc:HGNC:24547] | 489 | 0.662 | 0.0273 | No |
| 12 | NR0B2 | nuclear receptor subfamily 0 group B member 2 [Source:HGNC Symbol;Acc:HGNC:7961] | 573 | 0.632 | 0.0257 | No |
| 13 | KAT8 | lysine acetyltransferase 8 [Source:HGNC Symbol;Acc:HGNC:17933] | 595 | 0.625 | 0.0289 | No |
| 14 | ING4 | inhibitor of growth family member 4 [Source:HGNC Symbol;Acc:HGNC:19423] | 680 | 0.598 | 0.0269 | No |
| 15 | TRRAP | transformation/transcription domain associated protein [Source:HGNC Symbol;Acc:HGNC:12347] | 688 | 0.596 | 0.0310 | No |
| 16 | BRD7 | bromodomain containing 7 [Source:HGNC Symbol;Acc:HGNC:14310] | 726 | 0.588 | 0.0327 | No |
| 17 | HSBP1 | heat shock factor binding protein 1 [Source:HGNC Symbol;Acc:HGNC:5203] | 830 | 0.560 | 0.0289 | No |
| 18 | PHF10 | PHD finger protein 10 [Source:HGNC Symbol;Acc:HGNC:18250] | 842 | 0.557 | 0.0324 | No |
| 19 | DPF2 | double PHD fingers 2 [Source:HGNC Symbol;Acc:HGNC:9964] | 854 | 0.554 | 0.0359 | No |
| 20 | TRIM32 | tripartite motif containing 32 [Source:HGNC Symbol;Acc:HGNC:16380] | 993 | 0.519 | 0.0289 | No |
| 21 | PMF1 | polyamine modulated factor 1 [Source:HGNC Symbol;Acc:HGNC:9112] | 1059 | 0.505 | 0.0277 | No |
| 22 | TBL1XR1 | TBL1X receptor 1 [Source:HGNC Symbol;Acc:HGNC:29529] | 1070 | 0.501 | 0.0308 | No |
| 23 | SAP18 | Sin3A associated protein 18 [Source:HGNC Symbol;Acc:HGNC:10530] | 1099 | 0.496 | 0.0325 | No |
| 24 | KAT7 | lysine acetyltransferase 7 [Source:HGNC Symbol;Acc:HGNC:17016] | 1125 | 0.491 | 0.0344 | No |
| 25 | MAGED1 | MAGE family member D1 [Source:HGNC Symbol;Acc:HGNC:6813] | 1128 | 0.491 | 0.0381 | No |
| 26 | MED20 | mediator complex subunit 20 [Source:HGNC Symbol;Acc:HGNC:16840] | 1138 | 0.489 | 0.0412 | No |
| 27 | PRMT2 | protein arginine methyltransferase 2 [Source:HGNC Symbol;Acc:HGNC:5186] | 1171 | 0.482 | 0.0424 | No |
| 28 | TADA3 | transcriptional adaptor 3 [Source:HGNC Symbol;Acc:HGNC:19422] | 1295 | 0.459 | 0.0362 | No |
| 29 | URI1 | URI1 prefoldin like chaperone [Source:HGNC Symbol;Acc:HGNC:13236] | 1324 | 0.454 | 0.0375 | No |
| 30 | HDAC3 | histone deacetylase 3 [Source:HGNC Symbol;Acc:HGNC:4854] | 1325 | 0.454 | 0.0411 | No |
| 31 | WBP2 | WW domain binding protein 2 [Source:HGNC Symbol;Acc:HGNC:12738] | 1344 | 0.451 | 0.0432 | No |
| 32 | DCAF6 | DDB1 and CUL4 associated factor 6 [Source:HGNC Symbol;Acc:HGNC:30002] | 1357 | 0.449 | 0.0458 | No |
| 33 | BUD31 | BUD31 homolog [Source:HGNC Symbol;Acc:HGNC:29629] | 1380 | 0.446 | 0.0476 | No |
| 34 | CBX4 | chromobox 4 [Source:HGNC Symbol;Acc:HGNC:1554] | 1397 | 0.443 | 0.0498 | No |
| 35 | PARK7 | Parkinsonism associated deglycase [Source:HGNC Symbol;Acc:HGNC:16369] | 1406 | 0.442 | 0.0526 | No |
| 36 | NME2 | NME/NM23 nucleoside diphosphate kinase 2 [Source:HGNC Symbol;Acc:HGNC:7850] | 1428 | 0.439 | 0.0544 | No |
| 37 | HTATIP2 | HIV-1 Tat interactive protein 2 [Source:HGNC Symbol;Acc:HGNC:16637] | 1456 | 0.435 | 0.0556 | No |
| 38 | PSIP1 | PC4 and SFRS1 interacting protein 1 [Source:HGNC Symbol;Acc:HGNC:9527] | 1482 | 0.431 | 0.0570 | No |
| 39 | PSMD9 | "proteasome 26S subunit, non-ATPase 9 [Source:HGNC Symbol;Acc:HGNC:9567]" | 1576 | 0.417 | 0.0529 | No |
| 40 | WWOX | WW domain containing oxidoreductase [Source:HGNC Symbol;Acc:HGNC:12799] | 1614 | 0.411 | 0.0532 | No |
| 41 | UXT | ubiquitously expressed prefoldin like chaperone [Source:HGNC Symbol;Acc:HGNC:12641] | 1641 | 0.407 | 0.0543 | No |
| 42 | AIP | aryl hydrocarbon receptor interacting protein [Source:HGNC Symbol;Acc:HGNC:358] | 1649 | 0.406 | 0.0569 | No |
| 43 | CNOT7 | CCR4-NOT transcription complex subunit 7 [Source:HGNC Symbol;Acc:HGNC:14101] | 1676 | 0.402 | 0.0580 | No |
| 44 | EDF1 | endothelial differentiation related factor 1 [Source:HGNC Symbol;Acc:HGNC:3164] | 1684 | 0.401 | 0.0606 | No |
| 45 | MED6 | mediator complex subunit 6 [Source:HGNC Symbol;Acc:HGNC:19970] | 1744 | 0.393 | 0.0590 | No |
| 46 | BRDT | bromodomain testis associated [Source:HGNC Symbol;Acc:HGNC:1105] | 1812 | 0.382 | 0.0566 | No |
| 47 | C1D | C1D nuclear receptor corepressor [Source:HGNC Symbol;Acc:HGNC:29911] | 1848 | 0.379 | 0.0568 | No |
| 48 | TRIM21 | tripartite motif containing 21 [Source:HGNC Symbol;Acc:HGNC:11312] | 1937 | 0.368 | 0.0527 | No |
| 49 | CBFB | core-binding factor subunit beta [Source:HGNC Symbol;Acc:HGNC:1539] | 1962 | 0.366 | 0.0536 | No |
| 50 | PRKN | parkin RBR E3 ubiquitin protein ligase [Source:HGNC Symbol;Acc:HGNC:8607] | 1972 | 0.364 | 0.0558 | No |
| 51 | POU2AF1 | POU class 2 homeobox associating factor 1 [Source:HGNC Symbol;Acc:HGNC:9211] | 2002 | 0.360 | 0.0563 | No |
| 52 | TRIM8 | tripartite motif containing 8 [Source:HGNC Symbol;Acc:HGNC:15579] | 2063 | 0.355 | 0.0543 | No |
| 53 | TAF9B | TATA-box binding protein associated factor 9b [Source:HGNC Symbol;Acc:HGNC:17306] | 2081 | 0.352 | 0.0557 | No |
| 54 | ARRB1 | arrestin beta 1 [Source:HGNC Symbol;Acc:HGNC:711] | 2116 | 0.348 | 0.0557 | No |
| 55 | LMO2 | LIM domain only 2 [Source:HGNC Symbol;Acc:HGNC:6642] | 2122 | 0.347 | 0.0581 | No |
| 56 | ZNF410 | zinc finger protein 410 [Source:HGNC Symbol;Acc:HGNC:20144] | 2345 | 0.321 | 0.0428 | No |
| 57 | TCF25 | transcription factor 25 [Source:HGNC Symbol;Acc:HGNC:29181] | 2380 | 0.317 | 0.0426 | No |
| 58 | TRIM22 | tripartite motif containing 22 [Source:HGNC Symbol;Acc:HGNC:16379] | 2521 | 0.301 | 0.0338 | No |
| 59 | ACTN2 | actinin alpha 2 [Source:HGNC Symbol;Acc:HGNC:164] | 2532 | 0.299 | 0.0353 | No |
| 60 | PKN1 | protein kinase N1 [Source:HGNC Symbol;Acc:HGNC:9405] | 2540 | 0.298 | 0.0371 | No |
| 61 | NCOR1 | nuclear receptor corepressor 1 [Source:HGNC Symbol;Acc:HGNC:7672] | 2549 | 0.297 | 0.0388 | No |
| 62 | MED12 | mediator complex subunit 12 [Source:HGNC Symbol;Acc:HGNC:11957] | 2588 | 0.292 | 0.0381 | No |
| 63 | MED27 | mediator complex subunit 27 [Source:HGNC Symbol;Acc:HGNC:2377] | 2607 | 0.290 | 0.0389 | No |
| 64 | SS18 | SS18 subunit of BAF chromatin remodeling complex [Source:HGNC Symbol;Acc:HGNC:11340] | 2677 | 0.282 | 0.0356 | No |
| 65 | RUVBL2 | RuvB like AAA ATPase 2 [Source:HGNC Symbol;Acc:HGNC:10475] | 2778 | 0.272 | 0.0298 | No |
| 66 | VGLL1 | vestigial like family member 1 [Source:HGNC Symbol;Acc:HGNC:20985] | 2819 | 0.268 | 0.0287 | No |
| 67 | MED21 | mediator complex subunit 21 [Source:HGNC Symbol;Acc:HGNC:11473] | 2826 | 0.267 | 0.0303 | No |
| 68 | KAT6B | lysine acetyltransferase 6B [Source:HGNC Symbol;Acc:HGNC:17582] | 2827 | 0.267 | 0.0324 | No |
| 69 | BCOR | BCL6 corepressor [Source:HGNC Symbol;Acc:HGNC:20893] | 2890 | 0.262 | 0.0295 | No |
| 70 | MED31 | mediator complex subunit 31 [Source:HGNC Symbol;Acc:HGNC:24260] | 2904 | 0.260 | 0.0305 | No |
| 71 | ZBED1 | zinc finger BED-type containing 1 [Source:HGNC Symbol;Acc:HGNC:447] | 2912 | 0.259 | 0.0320 | No |
| 72 | LPIN2 | lipin 2 [Source:HGNC Symbol;Acc:HGNC:14450] | 2969 | 0.253 | 0.0295 | No |
| 73 | C1QBP | complement C1q binding protein [Source:HGNC Symbol;Acc:HGNC:1243] | 3083 | 0.241 | 0.0224 | No |
| 74 | SIAH2 | siah E3 ubiquitin protein ligase 2 [Source:HGNC Symbol;Acc:HGNC:10858] | 3147 | 0.235 | 0.0192 | No |
| 75 | CASP8AP2 | caspase 8 associated protein 2 [Source:HGNC Symbol;Acc:HGNC:1510] | 3186 | 0.232 | 0.0180 | No |
| 76 | LPXN | leupaxin [Source:HGNC Symbol;Acc:HGNC:14061] | 3232 | 0.227 | 0.0162 | No |
| 77 | MED24 | mediator complex subunit 24 [Source:HGNC Symbol;Acc:HGNC:22963] | 3246 | 0.226 | 0.0169 | No |
| 78 | MYCBP | MYC binding protein [Source:HGNC Symbol;Acc:HGNC:7554] | 3251 | 0.226 | 0.0184 | No |
| 79 | XPC | "XPC complex subunit, DNA damage recognition and repair factor [Source:HGNC Symbol;Acc:HGNC:12816]" | 3278 | 0.222 | 0.0180 | No |
| 80 | SMARCA2 | "SWI/SNF related, matrix associated, actin dependent regulator of chromatin, subfamily a, member 2 [Source:HGNC Symbol;Acc:HGNC:11098]" | 3281 | 0.222 | 0.0196 | No |
| 81 | DDX1 | DEAD-box helicase 1 [Source:HGNC Symbol;Acc:HGNC:2734] | 3305 | 0.221 | 0.0195 | No |
| 82 | SUPT3H | "SPT3 homolog, SAGA and STAGA complex component [Source:HGNC Symbol;Acc:HGNC:11466]" | 3340 | 0.218 | 0.0185 | No |
| 83 | HR | HR lysine demethylase and nuclear receptor corepressor [Source:HGNC Symbol;Acc:HGNC:5172] | 3356 | 0.216 | 0.0190 | No |
| 84 | ELK1 | ETS transcription factor ELK1 [Source:HGNC Symbol;Acc:HGNC:3321] | 3357 | 0.216 | 0.0207 | No |
| 85 | PCBD1 | pterin-4 alpha-carbinolamine dehydratase 1 [Source:HGNC Symbol;Acc:HGNC:8646] | 3405 | 0.211 | 0.0186 | No |
| 86 | TOX3 | TOX high mobility group box family member 3 [Source:HGNC Symbol;Acc:HGNC:11972] | 3438 | 0.207 | 0.0177 | No |
| 87 | RBFOX2 | RNA binding fox-1 homolog 2 [Source:HGNC Symbol;Acc:HGNC:9906] | 3523 | 0.200 | 0.0125 | No |
| 88 | TRIM28 | tripartite motif containing 28 [Source:HGNC Symbol;Acc:HGNC:16384] | 3526 | 0.200 | 0.0139 | No |
| 89 | RALY | RALY heterogeneous nuclear ribonucleoprotein [Source:HGNC Symbol;Acc:HGNC:15921] | 3546 | 0.198 | 0.0140 | No |
| 90 | ENY2 | ENY2 transcription and export complex 2 subunit [Source:HGNC Symbol;Acc:HGNC:24449] | 3561 | 0.197 | 0.0144 | No |
| 91 | EZH1 | enhancer of zeste 1 polycomb repressive complex 2 subunit [Source:HGNC Symbol;Acc:HGNC:3526] | 3565 | 0.197 | 0.0157 | No |
| 92 | TGFB1I1 | transforming growth factor beta 1 induced transcript 1 [Source:HGNC Symbol;Acc:HGNC:11767] | 3628 | 0.191 | 0.0123 | No |
| 93 | ZXDA | zinc finger X-linked duplicated A [Source:HGNC Symbol;Acc:HGNC:13198] | 3726 | 0.183 | 0.0060 | No |
| 94 | RBBP8 | "RB binding protein 8, endonuclease [Source:HGNC Symbol;Acc:HGNC:9891]" | 3739 | 0.182 | 0.0064 | No |
| 95 | USP16 | ubiquitin specific peptidase 16 [Source:HGNC Symbol;Acc:HGNC:12614] | 3831 | 0.173 | 0.0005 | No |
| 96 | TAF12 | TATA-box binding protein associated factor 12 [Source:HGNC Symbol;Acc:HGNC:11545] | 3843 | 0.172 | 0.0010 | No |
| 97 | RNF4 | ring finger protein 4 [Source:HGNC Symbol;Acc:HGNC:10067] | 3851 | 0.170 | 0.0018 | No |
| 98 | TRIM52 | tripartite motif containing 52 [Source:HGNC Symbol;Acc:HGNC:19024] | 3863 | 0.169 | 0.0022 | No |
| 99 | SND1 | staphylococcal nuclease and tudor domain containing 1 [Source:HGNC Symbol;Acc:HGNC:30646] | 3897 | 0.165 | 0.0009 | No |
| 100 | ZFPM2 | "zinc finger protein, FOG family member 2 [Source:HGNC Symbol;Acc:HGNC:16700]" | 3958 | 0.160 | -0.0027 | No |
| 101 | PFDN5 | prefoldin subunit 5 [Source:HGNC Symbol;Acc:HGNC:8869] | 3959 | 0.159 | -0.0014 | No |
| 102 | HDGFL3 | HDGF like 3 [Source:HGNC Symbol;Acc:HGNC:24937] | 3970 | 0.158 | -0.0010 | No |
| 103 | PEX14 | peroxisomal biogenesis factor 14 [Source:HGNC Symbol;Acc:HGNC:8856] | 3985 | 0.157 | -0.0009 | No |
| 104 | TRIP4 | thyroid hormone receptor interactor 4 [Source:HGNC Symbol;Acc:HGNC:12310] | 4105 | 0.146 | -0.0092 | No |
| 105 | TDP2 | tyrosyl-DNA phosphodiesterase 2 [Source:HGNC Symbol;Acc:HGNC:17768] | 4124 | 0.144 | -0.0095 | No |
| 106 | FHL2 | four and a half LIM domains 2 [Source:HGNC Symbol;Acc:HGNC:3703] | 4127 | 0.143 | -0.0086 | No |
| 107 | TRIP13 | thyroid hormone receptor interactor 13 [Source:HGNC Symbol;Acc:HGNC:12307] | 4184 | 0.139 | -0.0119 | No |
| 108 | SMARCE1 | "SWI/SNF related, matrix associated, actin dependent regulator of chromatin, subfamily e, member 1 [Source:HGNC Symbol;Acc:HGNC:11109]" | 4255 | 0.132 | -0.0165 | No |
| 109 | TAF6 | TATA-box binding protein associated factor 6 [Source:HGNC Symbol;Acc:HGNC:11540] | 4260 | 0.132 | -0.0158 | No |
| 110 | COPS5 | COP9 signalosome subunit 5 [Source:HGNC Symbol;Acc:HGNC:2240] | 4261 | 0.131 | -0.0148 | No |
| 111 | NCOA2 | nuclear receptor coactivator 2 [Source:HGNC Symbol;Acc:HGNC:7669] | 4284 | 0.129 | -0.0155 | No |
| 112 | TRIM13 | tripartite motif containing 13 [Source:HGNC Symbol;Acc:HGNC:9976] | 4441 | 0.118 | -0.0271 | No |
| 113 | DRAP1 | DR1 associated protein 1 [Source:HGNC Symbol;Acc:HGNC:3019] | 4462 | 0.116 | -0.0277 | No |
| 114 | COPS2 | COP9 signalosome subunit 2 [Source:HGNC Symbol;Acc:HGNC:30747] | 4494 | 0.113 | -0.0293 | No |
| 115 | SETD3 | "SET domain containing 3, actin histidine methyltransferase [Source:HGNC Symbol;Acc:HGNC:20493]" | 4506 | 0.113 | -0.0293 | No |
| 116 | PRPF6 | pre-mRNA processing factor 6 [Source:HGNC Symbol;Acc:HGNC:15860] | 4567 | 0.108 | -0.0333 | No |
| 117 | USP22 | ubiquitin specific peptidase 22 [Source:HGNC Symbol;Acc:HGNC:12621] | 4687 | 0.097 | -0.0420 | No |
| 118 | ABT1 | activator of basal transcription 1 [Source:HGNC Symbol;Acc:HGNC:17369] | 4783 | 0.088 | -0.0489 | No |
| 119 | KAT2B | lysine acetyltransferase 2B [Source:HGNC Symbol;Acc:HGNC:8638] | 4867 | 0.082 | -0.0549 | No |
| 120 | SAP30 | Sin3A associated protein 30 [Source:HGNC Symbol;Acc:HGNC:10532] | 4907 | 0.079 | -0.0574 | No |
| 121 | BASP1 | brain abundant membrane attached signal protein 1 [Source:HGNC Symbol;Acc:HGNC:957] | 4923 | 0.078 | -0.0580 | No |
| 122 | HCFC1 | host cell factor C1 [Source:HGNC Symbol;Acc:HGNC:4839] | 4937 | 0.077 | -0.0584 | No |
| 123 | TP53BP1 | tumor protein p53 binding protein 1 [Source:HGNC Symbol;Acc:HGNC:11999] | 4946 | 0.076 | -0.0585 | No |
| 124 | LDB2 | LIM domain binding 2 [Source:HGNC Symbol;Acc:HGNC:6533] | 5006 | 0.073 | -0.0626 | No |
| 125 | CDYL | chromodomain Y like [Source:HGNC Symbol;Acc:HGNC:1811] | 5016 | 0.072 | -0.0628 | No |
| 126 | SMARCD3 | "SWI/SNF related, matrix associated, actin dependent regulator of chromatin, subfamily d, member 3 [Source:HGNC Symbol;Acc:HGNC:11108]" | 5036 | 0.070 | -0.0638 | No |
| 127 | FHL5 | four and a half LIM domains 5 [Source:HGNC Symbol;Acc:HGNC:17371] | 5038 | 0.070 | -0.0633 | No |
| 128 | YAP1 | Yes1 associated transcriptional regulator [Source:HGNC Symbol;Acc:HGNC:16262] | 5041 | 0.070 | -0.0629 | No |
| 129 | ZMIZ2 | zinc finger MIZ-type containing 2 [Source:HGNC Symbol;Acc:HGNC:22229] | 5044 | 0.069 | -0.0625 | No |
| 130 | BIRC2 | baculoviral IAP repeat containing 2 [Source:HGNC Symbol;Acc:HGNC:590] | 5054 | 0.069 | -0.0627 | No |
| 131 | IRF4 | interferon regulatory factor 4 [Source:HGNC Symbol;Acc:HGNC:6119] | 5064 | 0.067 | -0.0629 | No |
| 132 | CTBP2 | C-terminal binding protein 2 [Source:HGNC Symbol;Acc:HGNC:2495] | 5066 | 0.067 | -0.0624 | No |
| 133 | PHB | prohibitin [Source:HGNC Symbol;Acc:HGNC:8912] | 5102 | 0.064 | -0.0647 | No |
| 134 | SAP30L | SAP30 like [Source:HGNC Symbol;Acc:HGNC:25663] | 5124 | 0.062 | -0.0659 | No |
| 135 | SMARCD2 | "SWI/SNF related, matrix associated, actin dependent regulator of chromatin, subfamily d, member 2 [Source:HGNC Symbol;Acc:HGNC:11107]" | 5129 | 0.062 | -0.0657 | No |
| 136 | NMI | N-myc and STAT interactor [Source:HGNC Symbol;Acc:HGNC:7854] | 5153 | 0.059 | -0.0671 | No |
| 137 | NSD1 | nuclear receptor binding SET domain protein 1 [Source:HGNC Symbol;Acc:HGNC:14234] | 5178 | 0.058 | -0.0686 | No |
| 138 | HDAC9 | histone deacetylase 9 [Source:HGNC Symbol;Acc:HGNC:14065] | 5183 | 0.058 | -0.0684 | No |
| 139 | PIAS3 | protein inhibitor of activated STAT 3 [Source:HGNC Symbol;Acc:HGNC:16861] | 5195 | 0.057 | -0.0689 | No |
| 140 | TRIP11 | thyroid hormone receptor interactor 11 [Source:HGNC Symbol;Acc:HGNC:12305] | 5233 | 0.054 | -0.0714 | No |
| 141 | MED17 | mediator complex subunit 17 [Source:HGNC Symbol;Acc:HGNC:2375] | 5257 | 0.052 | -0.0729 | No |
| 142 | MED22 | mediator complex subunit 22 [Source:HGNC Symbol;Acc:HGNC:11477] | 5259 | 0.051 | -0.0725 | No |
| 143 | HMGB2 | high mobility group box 2 [Source:HGNC Symbol;Acc:HGNC:5000] | 5272 | 0.050 | -0.0731 | No |
| 144 | PHF2 | PHD finger protein 2 [Source:HGNC Symbol;Acc:HGNC:8920] | 5332 | 0.045 | -0.0775 | No |
| 145 | TFAP2A | transcription factor AP-2 alpha [Source:HGNC Symbol;Acc:HGNC:11742] | 5379 | 0.041 | -0.0808 | No |
| 146 | UBE2L3 | ubiquitin conjugating enzyme E2 L3 [Source:HGNC Symbol;Acc:HGNC:12488] | 5428 | 0.038 | -0.0843 | No |
| 147 | NPAT | "nuclear protein, coactivator of histone transcription [Source:HGNC Symbol;Acc:HGNC:7896]" | 5432 | 0.038 | -0.0843 | No |
| 148 | SNW1 | SNW domain containing 1 [Source:HGNC Symbol;Acc:HGNC:16696] | 5472 | 0.035 | -0.0871 | No |
| 149 | TRIM27 | tripartite motif containing 27 [Source:HGNC Symbol;Acc:HGNC:9975] | 5478 | 0.034 | -0.0873 | No |
| 150 | KDM5B | lysine demethylase 5B [Source:HGNC Symbol;Acc:HGNC:18039] | 5484 | 0.033 | -0.0874 | No |
| 151 | SS18L2 | SS18 like 2 [Source:HGNC Symbol;Acc:HGNC:15593] | 5487 | 0.033 | -0.0873 | No |
| 152 | RUNX1T1 | RUNX1 partner transcriptional co-repressor 1 [Source:HGNC Symbol;Acc:HGNC:1535] | 5503 | 0.032 | -0.0882 | No |
| 153 | NUPR1 | "nuclear protein 1, transcriptional regulator [Source:HGNC Symbol;Acc:HGNC:29990]" | 5616 | 0.023 | -0.0970 | No |
| 154 | MED23 | mediator complex subunit 23 [Source:HGNC Symbol;Acc:HGNC:2372] | 5623 | 0.022 | -0.0973 | No |
| 155 | ARID1A | AT-rich interaction domain 1A [Source:HGNC Symbol;Acc:HGNC:11110] | 5735 | 0.013 | -0.1061 | No |
| 156 | CNOT2 | CCR4-NOT transcription complex subunit 2 [Source:HGNC Symbol;Acc:HGNC:7878] | 5740 | 0.012 | -0.1063 | No |
| 157 | PIR | pirin [Source:HGNC Symbol;Acc:HGNC:30048] | 5743 | 0.012 | -0.1064 | No |
| 158 | KAT6A | lysine acetyltransferase 6A [Source:HGNC Symbol;Acc:HGNC:13013] | 5778 | 0.010 | -0.1090 | No |
| 159 | PQBP1 | polyglutamine binding protein 1 [Source:HGNC Symbol;Acc:HGNC:9330] | 5827 | 0.007 | -0.1128 | No |
| 160 | MED14 | mediator complex subunit 14 [Source:HGNC Symbol;Acc:HGNC:2370] | 5867 | 0.004 | -0.1159 | No |
| 161 | SERTAD2 | SERTA domain containing 2 [Source:HGNC Symbol;Acc:HGNC:30784] | 5877 | 0.004 | -0.1166 | No |
| 162 | DPF3 | double PHD fingers 3 [Source:HGNC Symbol;Acc:HGNC:17427] | 5907 | 0.002 | -0.1189 | No |
| 163 | CBFA2T3 | CBFA2/RUNX1 partner transcriptional co-repressor 3 [Source:HGNC Symbol;Acc:HGNC:1537] | 5951 | -0.001 | -0.1223 | No |
| 164 | YAF2 | YY1 associated factor 2 [Source:HGNC Symbol;Acc:HGNC:17363] | 5995 | -0.004 | -0.1258 | No |
| 165 | KDM3B | lysine demethylase 3B [Source:HGNC Symbol;Acc:HGNC:1337] | 6005 | -0.005 | -0.1264 | No |
| 166 | RB1 | RB transcriptional corepressor 1 [Source:HGNC Symbol;Acc:HGNC:9884] | 6013 | -0.006 | -0.1270 | No |
| 167 | RCOR3 | REST corepressor 3 [Source:HGNC Symbol;Acc:HGNC:25594] | 6014 | -0.006 | -0.1269 | No |
| 168 | ARID3A | AT-rich interaction domain 3A [Source:HGNC Symbol;Acc:HGNC:3031] | 6031 | -0.007 | -0.1281 | No |
| 169 | USP21 | ubiquitin specific peptidase 21 [Source:HGNC Symbol;Acc:HGNC:12620] | 6096 | -0.012 | -0.1332 | No |
| 170 | ZMIZ1 | zinc finger MIZ-type containing 1 [Source:HGNC Symbol;Acc:HGNC:16493] | 6111 | -0.013 | -0.1342 | No |
| 171 | TRIM37 | tripartite motif containing 37 [Source:HGNC Symbol;Acc:HGNC:7523] | 6176 | -0.019 | -0.1392 | No |
| 172 | PPARGC1B | PPARG coactivator 1 beta [Source:HGNC Symbol;Acc:HGNC:30022] | 6184 | -0.019 | -0.1396 | No |
| 173 | SCAI | suppressor of cancer cell invasion [Source:HGNC Symbol;Acc:HGNC:26709] | 6199 | -0.020 | -0.1405 | No |
| 174 | MED16 | mediator complex subunit 16 [Source:HGNC Symbol;Acc:HGNC:17556] | 6240 | -0.023 | -0.1435 | No |
| 175 | TAF9 | TATA-box binding protein associated factor 9 [Source:HGNC Symbol;Acc:HGNC:11542] | 6297 | -0.027 | -0.1478 | No |
| 176 | WWC1 | WW and C2 domain containing 1 [Source:HGNC Symbol;Acc:HGNC:29435] | 6376 | -0.034 | -0.1538 | No |
| 177 | KAT5 | lysine acetyltransferase 5 [Source:HGNC Symbol;Acc:HGNC:5275] | 6379 | -0.034 | -0.1537 | No |
| 178 | NAB1 | NGFI-A binding protein 1 [Source:HGNC Symbol;Acc:HGNC:7626] | 6396 | -0.034 | -0.1547 | No |
| 179 | ANKRD1 | ankyrin repeat domain 1 [Source:HGNC Symbol;Acc:HGNC:15819] | 6416 | -0.037 | -0.1559 | No |
| 180 | PBXIP1 | PBX homeobox interacting protein 1 [Source:HGNC Symbol;Acc:HGNC:21199] | 6434 | -0.038 | -0.1570 | No |
| 181 | PRKCB | protein kinase C beta [Source:HGNC Symbol;Acc:HGNC:9395] | 6453 | -0.039 | -0.1581 | No |
| 182 | TRIM31 | tripartite motif containing 31 [Source:HGNC Symbol;Acc:HGNC:16289] | 6472 | -0.040 | -0.1592 | No |
| 183 | TRIM5 | tripartite motif containing 5 [Source:HGNC Symbol;Acc:HGNC:16276] | 6482 | -0.041 | -0.1596 | No |
| 184 | NCOR2 | nuclear receptor corepressor 2 [Source:HGNC Symbol;Acc:HGNC:7673] | 6497 | -0.042 | -0.1604 | No |
| 185 | EZH2 | enhancer of zeste 2 polycomb repressive complex 2 subunit [Source:HGNC Symbol;Acc:HGNC:3527] | 6562 | -0.047 | -0.1652 | No |
| 186 | HCFC2 | host cell factor C2 [Source:HGNC Symbol;Acc:HGNC:24972] | 6644 | -0.053 | -0.1712 | No |
| 187 | SSX1 | SSX family member 1 [Source:HGNC Symbol;Acc:HGNC:11335] | 6693 | -0.055 | -0.1746 | No |
| 188 | MMS19 | "MMS19 homolog, cytosolic iron-sulfur assembly component [Source:HGNC Symbol;Acc:HGNC:13824]" | 6733 | -0.058 | -0.1773 | No |
| 189 | HMGA2 | high mobility group AT-hook 2 [Source:HGNC Symbol;Acc:HGNC:5009] | 6850 | -0.067 | -0.1861 | No |
| 190 | ELANE | "elastase, neutrophil expressed [Source:HGNC Symbol;Acc:HGNC:3309]" | 6862 | -0.068 | -0.1864 | No |
| 191 | NR0B1 | nuclear receptor subfamily 0 group B member 1 [Source:HGNC Symbol;Acc:HGNC:7960] | 6885 | -0.070 | -0.1876 | No |
| 192 | HDGF | heparin binding growth factor [Source:HGNC Symbol;Acc:HGNC:4856] | 6924 | -0.074 | -0.1901 | No |
| 193 | JMJD1C | jumonji domain containing 1C [Source:HGNC Symbol;Acc:HGNC:12313] | 6989 | -0.079 | -0.1946 | No |
| 194 | SUPT20H | "SPT20 homolog, SAGA complex component [Source:HGNC Symbol;Acc:HGNC:20596]" | 6990 | -0.079 | -0.1939 | No |
| 195 | FOXP3 | forkhead box P3 [Source:HGNC Symbol;Acc:HGNC:6106] | 7035 | -0.082 | -0.1968 | No |
| 196 | FHL3 | four and a half LIM domains 3 [Source:HGNC Symbol;Acc:HGNC:3704] | 7061 | -0.085 | -0.1981 | No |
| 197 | CITED2 | Cbp/p300 interacting transactivator with Glu/Asp rich carboxy-terminal domain 2 [Source:HGNC Symbol;Acc:HGNC:1987] | 7100 | -0.087 | -0.2005 | No |
| 198 | TFB2M | "transcription factor B2, mitochondrial [Source:HGNC Symbol;Acc:HGNC:18559]" | 7112 | -0.089 | -0.2007 | No |
| 199 | NCOA6 | nuclear receptor coactivator 6 [Source:HGNC Symbol;Acc:HGNC:15936] | 7171 | -0.093 | -0.2046 | No |
| 200 | FGF2 | fibroblast growth factor 2 [Source:HGNC Symbol;Acc:HGNC:3676] | 7183 | -0.095 | -0.2047 | No |
| 201 | TLE2 | "TLE family member 2, transcriptional corepressor [Source:HGNC Symbol;Acc:HGNC:11838]" | 7198 | -0.096 | -0.2051 | No |
| 202 | MED4 | mediator complex subunit 4 [Source:HGNC Symbol;Acc:HGNC:17903] | 7236 | -0.098 | -0.2073 | No |
| 203 | MAML1 | mastermind like transcriptional coactivator 1 [Source:HGNC Symbol;Acc:HGNC:13632] | 7275 | -0.101 | -0.2095 | No |
| 204 | LIMD1 | LIM domain containing 1 [Source:HGNC Symbol;Acc:HGNC:6612] | 7281 | -0.101 | -0.2091 | No |
| 205 | PAGE4 | PAGE family member 4 [Source:HGNC Symbol;Acc:HGNC:4108] | 7292 | -0.102 | -0.2091 | No |
| 206 | ATF7IP | activating transcription factor 7 interacting protein [Source:HGNC Symbol;Acc:HGNC:20092] | 7386 | -0.109 | -0.2157 | No |
| 207 | RSF1 | remodeling and spacing factor 1 [Source:HGNC Symbol;Acc:HGNC:18118] | 7393 | -0.110 | -0.2153 | No |
| 208 | CAMTA1 | calmodulin binding transcription activator 1 [Source:HGNC Symbol;Acc:HGNC:18806] | 7400 | -0.111 | -0.2149 | No |
| 209 | ZXDB | zinc finger X-linked duplicated B [Source:HGNC Symbol;Acc:HGNC:13199] | 7435 | -0.114 | -0.2167 | No |
| 210 | RUVBL1 | RuvB like AAA ATPase 1 [Source:HGNC Symbol;Acc:HGNC:10474] | 7477 | -0.116 | -0.2191 | No |
| 211 | ZZEF1 | zinc finger ZZ-type and EF-hand domain containing 1 [Source:HGNC Symbol;Acc:HGNC:29027] | 7490 | -0.118 | -0.2191 | No |
| 212 | CCND1 | cyclin D1 [Source:HGNC Symbol;Acc:HGNC:1582] | 7512 | -0.119 | -0.2199 | No |
| 213 | CALCOCO1 | calcium binding and coiled-coil domain 1 [Source:HGNC Symbol;Acc:HGNC:29306] | 7559 | -0.124 | -0.2226 | No |
| 214 | PER2 | period circadian regulator 2 [Source:HGNC Symbol;Acc:HGNC:8846] | 7663 | -0.132 | -0.2298 | No |
| 215 | DYRK1A | dual specificity tyrosine phosphorylation regulated kinase 1A [Source:HGNC Symbol;Acc:HGNC:3091] | 7667 | -0.132 | -0.2290 | No |
| 216 | MID2 | midline 2 [Source:HGNC Symbol;Acc:HGNC:7096] | 7813 | -0.142 | -0.2395 | No |
| 217 | WWTR1 | WW domain containing transcription regulator 1 [Source:HGNC Symbol;Acc:HGNC:24042] | 7824 | -0.143 | -0.2392 | No |
| 218 | GMNN | geminin DNA replication inhibitor [Source:HGNC Symbol;Acc:HGNC:17493] | 7866 | -0.146 | -0.2413 | No |
| 219 | MUC1 | "mucin 1, cell surface associated [Source:HGNC Symbol;Acc:HGNC:7508]" | 7871 | -0.146 | -0.2405 | No |
| 220 | HSPA1A | heat shock protein family A (Hsp70) member 1A [Source:HGNC Symbol;Acc:HGNC:5232] | 7876 | -0.146 | -0.2396 | No |
| 221 | TOB1 | "transducer of ERBB2, 1 [Source:HGNC Symbol;Acc:HGNC:11979]" | 7892 | -0.147 | -0.2397 | No |
| 222 | PRMT5 | protein arginine methyltransferase 5 [Source:HGNC Symbol;Acc:HGNC:10894] | 7904 | -0.148 | -0.2394 | No |
| 223 | HDAC1 | histone deacetylase 1 [Source:HGNC Symbol;Acc:HGNC:4852] | 7911 | -0.148 | -0.2387 | No |
| 224 | TLE4 | "TLE family member 4, transcriptional corepressor [Source:HGNC Symbol;Acc:HGNC:11840]" | 7915 | -0.149 | -0.2378 | No |
| 225 | GON4L | gon-4 like [Source:HGNC Symbol;Acc:HGNC:25973] | 7935 | -0.150 | -0.2381 | No |
| 226 | PPP1R13L | protein phosphatase 1 regulatory subunit 13 like [Source:HGNC Symbol;Acc:HGNC:18838] | 7967 | -0.153 | -0.2394 | No |
| 227 | ACTL6B | actin like 6B [Source:HGNC Symbol;Acc:HGNC:160] | 8002 | -0.155 | -0.2409 | No |
| 228 | SMARCC1 | "SWI/SNF related, matrix associated, actin dependent regulator of chromatin subfamily c member 1 [Source:HGNC Symbol;Acc:HGNC:11104]" | 8052 | -0.159 | -0.2436 | No |
| 229 | CDY1 | chromodomain Y-linked 1 [Source:HGNC Symbol;Acc:HGNC:1809] | 8096 | -0.163 | -0.2457 | No |
| 230 | CARM1 | coactivator associated arginine methyltransferase 1 [Source:HGNC Symbol;Acc:HGNC:23393] | 8131 | -0.165 | -0.2471 | No |
| 231 | BCLAF1 | BCL2 associated transcription factor 1 [Source:HGNC Symbol;Acc:HGNC:16863] | 8193 | -0.170 | -0.2507 | No |
| 232 | SUB1 | SUB1 regulator of transcription [Source:HGNC Symbol;Acc:HGNC:19985] | 8200 | -0.171 | -0.2498 | No |
| 233 | KDM3A | lysine demethylase 3A [Source:HGNC Symbol;Acc:HGNC:20815] | 8264 | -0.176 | -0.2535 | No |
| 234 | DYRK1B | dual specificity tyrosine phosphorylation regulated kinase 1B [Source:HGNC Symbol;Acc:HGNC:3092] | 8346 | -0.182 | -0.2585 | No |
| 235 | ACTN1 | actinin alpha 1 [Source:HGNC Symbol;Acc:HGNC:163] | 8385 | -0.184 | -0.2601 | No |
| 236 | BRCA1 | BRCA1 DNA repair associated [Source:HGNC Symbol;Acc:HGNC:1100] | 8417 | -0.187 | -0.2611 | No |
| 237 | TRIM38 | tripartite motif containing 38 [Source:HGNC Symbol;Acc:HGNC:10059] | 8436 | -0.189 | -0.2611 | No |
| 238 | SMARCC2 | "SWI/SNF related, matrix associated, actin dependent regulator of chromatin subfamily c member 2 [Source:HGNC Symbol;Acc:HGNC:11105]" | 8480 | -0.192 | -0.2630 | No |
| 239 | LDB1 | LIM domain binding 1 [Source:HGNC Symbol;Acc:HGNC:6532] | 8522 | -0.195 | -0.2647 | No |
| 240 | NRIP1 | nuclear receptor interacting protein 1 [Source:HGNC Symbol;Acc:HGNC:8001] | 8524 | -0.195 | -0.2633 | No |
| 241 | SIRT6 | sirtuin 6 [Source:HGNC Symbol;Acc:HGNC:14934] | 8532 | -0.196 | -0.2623 | No |
| 242 | RBPMS | "RNA binding protein, mRNA processing factor [Source:HGNC Symbol;Acc:HGNC:19097]" | 8547 | -0.197 | -0.2619 | No |
| 243 | GTF2A1L | general transcription factor IIA subunit 1 like [Source:HGNC Symbol;Acc:HGNC:30727] | 8548 | -0.197 | -0.2603 | No |
| 244 | KDM1A | lysine demethylase 1A [Source:HGNC Symbol;Acc:HGNC:29079] | 8608 | -0.202 | -0.2634 | No |
| 245 | ASXL1 | ASXL transcriptional regulator 1 [Source:HGNC Symbol;Acc:HGNC:18318] | 8610 | -0.202 | -0.2619 | No |
| 246 | ATF7IP2 | activating transcription factor 7 interacting protein 2 [Source:HGNC Symbol;Acc:HGNC:20397] | 8636 | -0.203 | -0.2623 | No |
| 247 | MECP2 | methyl-CpG binding protein 2 [Source:HGNC Symbol;Acc:HGNC:6990] | 8687 | -0.208 | -0.2647 | No |
| 248 | YY1AP1 | YY1 associated protein 1 [Source:HGNC Symbol;Acc:HGNC:30935] | 8722 | -0.210 | -0.2657 | No |
| 249 | SIRT7 | sirtuin 7 [Source:HGNC Symbol;Acc:HGNC:14935] | 8733 | -0.211 | -0.2649 | No |
| 250 | RERE | arginine-glutamic acid dipeptide repeats [Source:HGNC Symbol;Acc:HGNC:9965] | 8744 | -0.212 | -0.2640 | No |
| 251 | MED8 | mediator complex subunit 8 [Source:HGNC Symbol;Acc:HGNC:19971] | 8751 | -0.212 | -0.2628 | No |
| 252 | UBE3A | ubiquitin protein ligase E3A [Source:HGNC Symbol;Acc:HGNC:12496] | 8757 | -0.213 | -0.2615 | No |
| 253 | PAWR | pro-apoptotic WT1 regulator [Source:HGNC Symbol;Acc:HGNC:8614] | 8766 | -0.213 | -0.2605 | No |
| 254 | APEX1 | apurinic/apyrimidinic endodeoxyribonuclease 1 [Source:HGNC Symbol;Acc:HGNC:587] | 8767 | -0.213 | -0.2588 | No |
| 255 | CREG1 | cellular repressor of E1A stimulated genes 1 [Source:HGNC Symbol;Acc:HGNC:2351] | 8770 | -0.214 | -0.2573 | No |
| 256 | TAF11 | TATA-box binding protein associated factor 11 [Source:HGNC Symbol;Acc:HGNC:11544] | 8860 | -0.222 | -0.2627 | No |
| 257 | TLE1 | "TLE family member 1, transcriptional corepressor [Source:HGNC Symbol;Acc:HGNC:11837]" | 8895 | -0.224 | -0.2636 | No |
| 258 | RRP1B | ribosomal RNA processing 1B [Source:HGNC Symbol;Acc:HGNC:23818] | 8909 | -0.225 | -0.2629 | No |
| 259 | DAXX | death domain associated protein [Source:HGNC Symbol;Acc:HGNC:2681] | 8926 | -0.226 | -0.2624 | No |
| 260 | SIRT1 | sirtuin 1 [Source:HGNC Symbol;Acc:HGNC:14929] | 8947 | -0.227 | -0.2622 | No |
| 261 | ZMYND11 | zinc finger MYND-type containing 11 [Source:HGNC Symbol;Acc:HGNC:16966] | 8952 | -0.228 | -0.2607 | No |
| 262 | LPIN1 | lipin 1 [Source:HGNC Symbol;Acc:HGNC:13345] | 9032 | -0.234 | -0.2652 | No |
| 263 | TDRD3 | tudor domain containing 3 [Source:HGNC Symbol;Acc:HGNC:20612] | 9080 | -0.238 | -0.2671 | No |
| 264 | ABL1 | "ABL proto-oncogene 1, non-receptor tyrosine kinase [Source:HGNC Symbol;Acc:HGNC:76]" | 9081 | -0.238 | -0.2652 | No |
| 265 | RCOR1 | REST corepressor 1 [Source:HGNC Symbol;Acc:HGNC:17441] | 9120 | -0.241 | -0.2663 | No |
| 266 | TLE3 | "TLE family member 3, transcriptional corepressor [Source:HGNC Symbol;Acc:HGNC:11839]" | 9121 | -0.241 | -0.2644 | No |
| 267 | MED9 | mediator complex subunit 9 [Source:HGNC Symbol;Acc:HGNC:25487] | 9146 | -0.244 | -0.2644 | No |
| 268 | PDLIM1 | PDZ and LIM domain 1 [Source:HGNC Symbol;Acc:HGNC:2067] | 9183 | -0.246 | -0.2654 | No |
| 269 | CIITA | class II major histocompatibility complex transactivator [Source:HGNC Symbol;Acc:HGNC:7067] | 9184 | -0.246 | -0.2634 | No |
| 270 | NRG1 | neuregulin 1 [Source:HGNC Symbol;Acc:HGNC:7997] | 9209 | -0.248 | -0.2634 | No |
| 271 | LCOR | ligand dependent nuclear receptor corepressor [Source:HGNC Symbol;Acc:HGNC:29503] | 9227 | -0.250 | -0.2628 | No |
| 272 | BCL10 | BCL10 immune signaling adaptor [Source:HGNC Symbol;Acc:HGNC:989] | 9248 | -0.252 | -0.2624 | No |
| 273 | NCOA1 | nuclear receptor coactivator 1 [Source:HGNC Symbol;Acc:HGNC:7668] | 9270 | -0.253 | -0.2621 | No |
| 274 | CBFA2T2 | CBFA2/RUNX1 partner transcriptional co-repressor 2 [Source:HGNC Symbol;Acc:HGNC:1536] | 9278 | -0.254 | -0.2607 | No |
| 275 | GPS2 | G protein pathway suppressor 2 [Source:HGNC Symbol;Acc:HGNC:4550] | 9338 | -0.258 | -0.2633 | No |
| 276 | DNAJB1 | DnaJ heat shock protein family (Hsp40) member B1 [Source:HGNC Symbol;Acc:HGNC:5270] | 9380 | -0.262 | -0.2646 | No |
| 277 | TBL1Y | transducin beta like 1 Y-linked [Source:HGNC Symbol;Acc:HGNC:18502] | 9416 | -0.266 | -0.2653 | No |
| 278 | LMCD1 | LIM and cysteine rich domains 1 [Source:HGNC Symbol;Acc:HGNC:6633] | 9464 | -0.270 | -0.2669 | No |
| 279 | BCL9 | BCL9 transcription coactivator [Source:HGNC Symbol;Acc:HGNC:1008] | 9587 | -0.282 | -0.2744 | No |
| 280 | NCOA4 | nuclear receptor coactivator 4 [Source:HGNC Symbol;Acc:HGNC:7671] | 9589 | -0.282 | -0.2723 | No |
| 281 | ENO1 | enolase 1 [Source:HGNC Symbol;Acc:HGNC:3350] | 9606 | -0.284 | -0.2713 | No |
| 282 | PSMC3IP | PSMC3 interacting protein [Source:HGNC Symbol;Acc:HGNC:17928] | 9612 | -0.284 | -0.2695 | No |
| 283 | CIR1 | "corepressor interacting with RBPJ, CIR1 [Source:HGNC Symbol;Acc:HGNC:24217]" | 9640 | -0.287 | -0.2694 | No |
| 284 | RBM14 | RNA binding motif protein 14 [Source:HGNC Symbol;Acc:HGNC:14219] | 9663 | -0.288 | -0.2689 | No |
| 285 | TACC1 | transforming acidic coiled-coil containing protein 1 [Source:HGNC Symbol;Acc:HGNC:11522] | 9690 | -0.290 | -0.2687 | No |
| 286 | TRIM62 | tripartite motif containing 62 [Source:HGNC Symbol;Acc:HGNC:25574] | 9737 | -0.295 | -0.2701 | No |
| 287 | JADE1 | jade family PHD finger 1 [Source:HGNC Symbol;Acc:HGNC:30027] | 9760 | -0.297 | -0.2695 | No |
| 288 | ETS1 | "ETS proto-oncogene 1, transcription factor [Source:HGNC Symbol;Acc:HGNC:3488]" | 9795 | -0.301 | -0.2698 | No |
| 289 | KAT2A | lysine acetyltransferase 2A [Source:HGNC Symbol;Acc:HGNC:4201] | 9799 | -0.301 | -0.2677 | No |
| 290 | PXN | paxillin [Source:HGNC Symbol;Acc:HGNC:9718] | 9845 | -0.305 | -0.2689 | No |
| 291 | HNRNPU | heterogeneous nuclear ribonucleoprotein U [Source:HGNC Symbol;Acc:HGNC:5048] | 9847 | -0.306 | -0.2666 | No |
| 292 | TRIM24 | tripartite motif containing 24 [Source:HGNC Symbol;Acc:HGNC:11812] | 9927 | -0.314 | -0.2704 | No |
| 293 | HMGA1 | high mobility group AT-hook 1 [Source:HGNC Symbol;Acc:HGNC:5010] | 9966 | -0.318 | -0.2709 | No |
| 294 | SMARCA4 | "SWI/SNF related, matrix associated, actin dependent regulator of chromatin, subfamily a, member 4 [Source:HGNC Symbol;Acc:HGNC:11100]" | 10009 | -0.322 | -0.2718 | No |
| 295 | DNMT3B | DNA methyltransferase 3 beta [Source:HGNC Symbol;Acc:HGNC:2979] | 10089 | -0.330 | -0.2755 | Yes |
| 296 | SFMBT1 | Scm like with four mbt domains 1 [Source:HGNC Symbol;Acc:HGNC:20255] | 10094 | -0.330 | -0.2732 | Yes |
| 297 | JUP | junction plakoglobin [Source:HGNC Symbol;Acc:HGNC:6207] | 10101 | -0.331 | -0.2711 | Yes |
| 298 | SMARCB1 | "SWI/SNF related, matrix associated, actin dependent regulator of chromatin, subfamily b, member 1 [Source:HGNC Symbol;Acc:HGNC:11103]" | 10117 | -0.333 | -0.2697 | Yes |
| 299 | TRIM15 | tripartite motif containing 15 [Source:HGNC Symbol;Acc:HGNC:16284] | 10156 | -0.337 | -0.2700 | Yes |
| 300 | CTBP1 | C-terminal binding protein 1 [Source:HGNC Symbol;Acc:HGNC:2494] | 10188 | -0.339 | -0.2699 | Yes |
| 301 | NAB2 | NGFI-A binding protein 2 [Source:HGNC Symbol;Acc:HGNC:7627] | 10196 | -0.340 | -0.2677 | Yes |
| 302 | PRDM16 | PR/SET domain 16 [Source:HGNC Symbol;Acc:HGNC:14000] | 10211 | -0.342 | -0.2662 | Yes |
| 303 | MAK | male germ cell associated kinase [Source:HGNC Symbol;Acc:HGNC:6816] | 10237 | -0.345 | -0.2654 | Yes |
| 304 | TLE5 | "TLE family member 5, transcriptional modulator [Source:HGNC Symbol;Acc:HGNC:307]" | 10253 | -0.346 | -0.2639 | Yes |
| 305 | CENPJ | centromere protein J [Source:HGNC Symbol;Acc:HGNC:17272] | 10271 | -0.348 | -0.2625 | Yes |
| 306 | RYBP | RING1 and YY1 binding protein [Source:HGNC Symbol;Acc:HGNC:10480] | 10386 | -0.359 | -0.2688 | Yes |
| 307 | HYAL2 | hyaluronidase 2 [Source:HGNC Symbol;Acc:HGNC:5321] | 10420 | -0.363 | -0.2686 | Yes |
| 308 | ACTN4 | actinin alpha 4 [Source:HGNC Symbol;Acc:HGNC:166] | 10472 | -0.370 | -0.2698 | Yes |
| 309 | WDR77 | WD repeat domain 77 [Source:HGNC Symbol;Acc:HGNC:29652] | 10489 | -0.372 | -0.2681 | Yes |
| 310 | PPARGC1A | PPARG coactivator 1 alpha [Source:HGNC Symbol;Acc:HGNC:9237] | 10504 | -0.374 | -0.2663 | Yes |
| 311 | SRSF2 | serine and arginine rich splicing factor 2 [Source:HGNC Symbol;Acc:HGNC:10783] | 10507 | -0.374 | -0.2635 | Yes |
| 312 | PA2G4 | proliferation-associated 2G4 [Source:HGNC Symbol;Acc:HGNC:8550] | 10598 | -0.383 | -0.2677 | Yes |
| 313 | MTA2 | metastasis associated 1 family member 2 [Source:HGNC Symbol;Acc:HGNC:7411] | 10600 | -0.383 | -0.2648 | Yes |
| 314 | ZNF451 | zinc finger protein 451 [Source:HGNC Symbol;Acc:HGNC:21091] | 10601 | -0.383 | -0.2617 | Yes |
| 315 | DHX9 | DExH-box helicase 9 [Source:HGNC Symbol;Acc:HGNC:2750] | 10627 | -0.386 | -0.2607 | Yes |
| 316 | TDG | thymine DNA glycosylase [Source:HGNC Symbol;Acc:HGNC:11700] | 10728 | -0.396 | -0.2656 | Yes |
| 317 | ACTL6A | actin like 6A [Source:HGNC Symbol;Acc:HGNC:24124] | 10821 | -0.407 | -0.2697 | Yes |
| 318 | TRIB3 | tribbles pseudokinase 3 [Source:HGNC Symbol;Acc:HGNC:16228] | 10839 | -0.409 | -0.2679 | Yes |
| 319 | NUP98 | nucleoporin 98 and 96 precursor [Source:HGNC Symbol;Acc:HGNC:8068] | 10875 | -0.413 | -0.2674 | Yes |
| 320 | CREBBP | CREB binding protein [Source:HGNC Symbol;Acc:HGNC:2348] | 10881 | -0.414 | -0.2646 | Yes |
| 321 | TAF5L | TATA-box binding protein associated factor 5 like [Source:HGNC Symbol;Acc:HGNC:17304] | 10906 | -0.418 | -0.2632 | Yes |
| 322 | KDM5A | lysine demethylase 5A [Source:HGNC Symbol;Acc:HGNC:9886] | 11097 | -0.443 | -0.2749 | Yes |
| 323 | MED1 | mediator complex subunit 1 [Source:HGNC Symbol;Acc:HGNC:9234] | 11146 | -0.450 | -0.2752 | Yes |
| 324 | MYBBP1A | MYB binding protein 1a [Source:HGNC Symbol;Acc:HGNC:7546] | 11188 | -0.456 | -0.2749 | Yes |
| 325 | UTF1 | undifferentiated embryonic cell transcription factor 1 [Source:HGNC Symbol;Acc:HGNC:12634] | 11200 | -0.457 | -0.2722 | Yes |
| 326 | CNOT6 | CCR4-NOT transcription complex subunit 6 [Source:HGNC Symbol;Acc:HGNC:14099] | 11212 | -0.459 | -0.2694 | Yes |
| 327 | SS18L1 | SS18L1 subunit of BAF chromatin remodeling complex [Source:HGNC Symbol;Acc:HGNC:15592] | 11261 | -0.466 | -0.2696 | Yes |
| 328 | TBL1X | transducin beta like 1 X-linked [Source:HGNC Symbol;Acc:HGNC:11585] | 11263 | -0.466 | -0.2660 | Yes |
| 329 | BTG1 | BTG anti-proliferation factor 1 [Source:HGNC Symbol;Acc:HGNC:1130] | 11267 | -0.466 | -0.2626 | Yes |
| 330 | N4BP2L2 | NEDD4 binding protein 2 like 2 [Source:HGNC Symbol;Acc:HGNC:26916] | 11271 | -0.467 | -0.2592 | Yes |
| 331 | NFKBIB | NFKB inhibitor beta [Source:HGNC Symbol;Acc:HGNC:7798] | 11277 | -0.468 | -0.2559 | Yes |
| 332 | PIAS2 | protein inhibitor of activated STAT 2 [Source:HGNC Symbol;Acc:HGNC:17311] | 11288 | -0.470 | -0.2530 | Yes |
| 333 | MED18 | mediator complex subunit 18 [Source:HGNC Symbol;Acc:HGNC:25944] | 11324 | -0.474 | -0.2520 | Yes |
| 334 | TMF1 | TATA element modulatory factor 1 [Source:HGNC Symbol;Acc:HGNC:11870] | 11328 | -0.475 | -0.2485 | Yes |
| 335 | MIER2 | MIER family member 2 [Source:HGNC Symbol;Acc:HGNC:29210] | 11375 | -0.483 | -0.2484 | Yes |
| 336 | PIAS1 | protein inhibitor of activated STAT 1 [Source:HGNC Symbol;Acc:HGNC:2752] | 11422 | -0.490 | -0.2482 | Yes |
| 337 | BCL3 | BCL3 transcription coactivator [Source:HGNC Symbol;Acc:HGNC:998] | 11431 | -0.493 | -0.2450 | Yes |
| 338 | RIOX2 | ribosomal oxygenase 2 [Source:HGNC Symbol;Acc:HGNC:19441] | 11444 | -0.495 | -0.2421 | Yes |
| 339 | EWSR1 | EWS RNA binding protein 1 [Source:HGNC Symbol;Acc:HGNC:3508] | 11445 | -0.495 | -0.2382 | Yes |
| 340 | BTAF1 | B-TFIID TATA-box binding protein associated factor 1 [Source:HGNC Symbol;Acc:HGNC:17307] | 11480 | -0.499 | -0.2370 | Yes |
| 341 | SUPT7L | "SPT7 like, STAGA complex subunit gamma [Source:HGNC Symbol;Acc:HGNC:30632]" | 11540 | -0.506 | -0.2377 | Yes |
| 342 | BRD8 | bromodomain containing 8 [Source:HGNC Symbol;Acc:HGNC:19874] | 11557 | -0.508 | -0.2350 | Yes |
| 343 | KMT2D | lysine methyltransferase 2D [Source:HGNC Symbol;Acc:HGNC:7133] | 11590 | -0.514 | -0.2335 | Yes |
| 344 | PHF8 | PHD finger protein 8 [Source:HGNC Symbol;Acc:HGNC:20672] | 11592 | -0.514 | -0.2295 | Yes |
| 345 | MRTFA | myocardin related transcription factor A [Source:HGNC Symbol;Acc:HGNC:14334] | 11613 | -0.518 | -0.2270 | Yes |
| 346 | SF1 | splicing factor 1 [Source:HGNC Symbol;Acc:HGNC:12950] | 11664 | -0.527 | -0.2269 | Yes |
| 347 | MRTFB | myocardin related transcription factor B [Source:HGNC Symbol;Acc:HGNC:29819] | 11668 | -0.527 | -0.2230 | Yes |
| 348 | ATN1 | atrophin 1 [Source:HGNC Symbol;Acc:HGNC:3033] | 11682 | -0.529 | -0.2199 | Yes |
| 349 | DDX17 | DEAD-box helicase 17 [Source:HGNC Symbol;Acc:HGNC:2740] | 11716 | -0.535 | -0.2183 | Yes |
| 350 | MED15 | mediator complex subunit 15 [Source:HGNC Symbol;Acc:HGNC:14248] | 11729 | -0.538 | -0.2150 | Yes |
| 351 | TCP10L | t-complex 10 like [Source:HGNC Symbol;Acc:HGNC:11657] | 11731 | -0.538 | -0.2108 | Yes |
| 352 | EP300 | E1A binding protein p300 [Source:HGNC Symbol;Acc:HGNC:3373] | 11741 | -0.540 | -0.2073 | Yes |
| 353 | MAP3K10 | mitogen-activated protein kinase kinase kinase 10 [Source:HGNC Symbol;Acc:HGNC:6849] | 11753 | -0.541 | -0.2039 | Yes |
| 354 | PTPN14 | protein tyrosine phosphatase non-receptor type 14 [Source:HGNC Symbol;Acc:HGNC:9647] | 11797 | -0.548 | -0.2031 | Yes |
| 355 | TRIM25 | tripartite motif containing 25 [Source:HGNC Symbol;Acc:HGNC:12932] | 11834 | -0.556 | -0.2016 | Yes |
| 356 | BTG2 | BTG anti-proliferation factor 2 [Source:HGNC Symbol;Acc:HGNC:1131] | 11917 | -0.576 | -0.2036 | Yes |
| 357 | NUCKS1 | nuclear casein kinase and cyclin dependent kinase substrate 1 [Source:HGNC Symbol;Acc:HGNC:29923] | 11945 | -0.581 | -0.2012 | Yes |
| 358 | TCERG1 | transcription elongation regulator 1 [Source:HGNC Symbol;Acc:HGNC:15630] | 11950 | -0.583 | -0.1969 | Yes |
| 359 | DDX54 | DEAD-box helicase 54 [Source:HGNC Symbol;Acc:HGNC:20084] | 11969 | -0.587 | -0.1937 | Yes |
| 360 | SMARCD1 | "SWI/SNF related, matrix associated, actin dependent regulator of chromatin, subfamily d, member 1 [Source:HGNC Symbol;Acc:HGNC:11106]" | 11991 | -0.592 | -0.1907 | Yes |
| 361 | KDM2A | lysine demethylase 2A [Source:HGNC Symbol;Acc:HGNC:13606] | 12011 | -0.595 | -0.1876 | Yes |
| 362 | PIAS4 | protein inhibitor of activated STAT 4 [Source:HGNC Symbol;Acc:HGNC:17002] | 12060 | -0.610 | -0.1866 | Yes |
| 363 | SPEN | spen family transcriptional repressor [Source:HGNC Symbol;Acc:HGNC:17575] | 12108 | -0.622 | -0.1855 | Yes |
| 364 | HIRA | histone cell cycle regulator [Source:HGNC Symbol;Acc:HGNC:4916] | 12143 | -0.630 | -0.1832 | Yes |
| 365 | HMGB1 | high mobility group box 1 [Source:HGNC Symbol;Acc:HGNC:4983] | 12170 | -0.636 | -0.1803 | Yes |
| 366 | ARID5B | AT-rich interaction domain 5B [Source:HGNC Symbol;Acc:HGNC:17362] | 12187 | -0.640 | -0.1765 | Yes |
| 367 | ZMYND8 | zinc finger MYND-type containing 8 [Source:HGNC Symbol;Acc:HGNC:9397] | 12214 | -0.649 | -0.1735 | Yes |
| 368 | TADA2A | transcriptional adaptor 2A [Source:HGNC Symbol;Acc:HGNC:11531] | 12217 | -0.650 | -0.1686 | Yes |
| 369 | NOC2L | NOC2 like nucleolar associated transcriptional repressor [Source:HGNC Symbol;Acc:HGNC:24517] | 12258 | -0.663 | -0.1665 | Yes |
| 370 | ZXDC | ZXD family zinc finger C [Source:HGNC Symbol;Acc:HGNC:28160] | 12259 | -0.663 | -0.1613 | Yes |
| 371 | TAF6L | TATA-box binding protein associated factor 6 like [Source:HGNC Symbol;Acc:HGNC:17305] | 12263 | -0.667 | -0.1563 | Yes |
| 372 | WNT4 | Wnt family member 4 [Source:HGNC Symbol;Acc:HGNC:12783] | 12331 | -0.686 | -0.1563 | Yes |
| 373 | IRF2BP1 | interferon regulatory factor 2 binding protein 1 [Source:HGNC Symbol;Acc:HGNC:21728] | 12387 | -0.704 | -0.1551 | Yes |
| 374 | KDM7A | lysine demethylase 7A [Source:HGNC Symbol;Acc:HGNC:22224] | 12390 | -0.704 | -0.1497 | Yes |
| 375 | AKIRIN1 | akirin 1 [Source:HGNC Symbol;Acc:HGNC:25744] | 12407 | -0.709 | -0.1454 | Yes |
| 376 | THRAP3 | thyroid hormone receptor associated protein 3 [Source:HGNC Symbol;Acc:HGNC:22964] | 12426 | -0.717 | -0.1412 | Yes |
| 377 | NCOA3 | nuclear receptor coactivator 3 [Source:HGNC Symbol;Acc:HGNC:7670] | 12455 | -0.727 | -0.1377 | Yes |
| 378 | CITED1 | Cbp/p300 interacting transactivator with Glu/Asp rich carboxy-terminal domain 1 [Source:HGNC Symbol;Acc:HGNC:1986] | 12493 | -0.746 | -0.1348 | Yes |
| 379 | MED13 | mediator complex subunit 13 [Source:HGNC Symbol;Acc:HGNC:22474] | 12504 | -0.749 | -0.1297 | Yes |
| 380 | NPM1 | nucleophosmin 1 [Source:HGNC Symbol;Acc:HGNC:7910] | 12530 | -0.762 | -0.1257 | Yes |
| 381 | SRCAP | Snf2 related CREBBP activator protein [Source:HGNC Symbol;Acc:HGNC:16974] | 12542 | -0.769 | -0.1206 | Yes |
| 382 | NOTCH1 | notch receptor 1 [Source:HGNC Symbol;Acc:HGNC:7881] | 12555 | -0.776 | -0.1154 | Yes |
| 383 | BRD4 | bromodomain containing 4 [Source:HGNC Symbol;Acc:HGNC:13575] | 12606 | -0.800 | -0.1131 | Yes |
| 384 | MTDH | metadherin [Source:HGNC Symbol;Acc:HGNC:29608] | 12621 | -0.809 | -0.1079 | Yes |
| 385 | CNOT9 | CCR4-NOT transcription complex subunit 9 [Source:HGNC Symbol;Acc:HGNC:10445] | 12668 | -0.839 | -0.1049 | Yes |
| 386 | MED13L | mediator complex subunit 13L [Source:HGNC Symbol;Acc:HGNC:22962] | 12670 | -0.844 | -0.0984 | Yes |
| 387 | DPF1 | double PHD fingers 1 [Source:HGNC Symbol;Acc:HGNC:20225] | 12671 | -0.844 | -0.0917 | Yes |
| 388 | PPRC1 | PPARG related coactivator 1 [Source:HGNC Symbol;Acc:HGNC:30025] | 12698 | -0.863 | -0.0870 | Yes |
| 389 | ARID5A | AT-rich interaction domain 5A [Source:HGNC Symbol;Acc:HGNC:17361] | 12705 | -0.867 | -0.0807 | Yes |
| 390 | FUS | FUS RNA binding protein [Source:HGNC Symbol;Acc:HGNC:4010] | 12739 | -0.902 | -0.0762 | Yes |
| 391 | MTA1 | metastasis associated 1 [Source:HGNC Symbol;Acc:HGNC:7410] | 12792 | -0.952 | -0.0729 | Yes |
| 392 | CEBPZ | CCAAT enhancer binding protein zeta [Source:HGNC Symbol;Acc:HGNC:24218] | 12796 | -0.961 | -0.0655 | Yes |
| 393 | AASS | aminoadipate-semialdehyde synthase [Source:HGNC Symbol;Acc:HGNC:17366] | 12799 | -0.968 | -0.0581 | Yes |
| 394 | CRYM | crystallin mu [Source:HGNC Symbol;Acc:HGNC:2418] | 12808 | -0.984 | -0.0510 | Yes |
| 395 | SIN3B | SIN3 transcription regulator family member B [Source:HGNC Symbol;Acc:HGNC:19354] | 12809 | -0.984 | -0.0432 | Yes |
| 396 | APBB1 | amyloid beta precursor protein binding family B member 1 [Source:HGNC Symbol;Acc:HGNC:581] | 12811 | -0.992 | -0.0355 | Yes |
| 397 | TAF15 | TATA-box binding protein associated factor 15 [Source:HGNC Symbol;Acc:HGNC:11547] | 12828 | -1.023 | -0.0287 | Yes |
| 398 | TOB2 | "transducer of ERBB2, 2 [Source:HGNC Symbol;Acc:HGNC:11980]" | 12838 | -1.041 | -0.0212 | Yes |
| 399 | HIPK2 | homeodomain interacting protein kinase 2 [Source:HGNC Symbol;Acc:HGNC:14402] | 12842 | -1.045 | -0.0132 | Yes |
| 400 | CTNNB1 | catenin beta 1 [Source:HGNC Symbol;Acc:HGNC:2514] | 12866 | -1.119 | -0.0063 | Yes |
| 401 | HDAC7 | histone deacetylase 7 [Source:HGNC Symbol;Acc:HGNC:14067] | 12870 | -1.131 | 0.0024 | Yes |
Table: GSEA details [plain text format]

  

Fig 2: GOMF\_TRANSCRIPTION\_COREGULATOR\_ACTIVITY      
 Blue-Pink O' Gram in the Space of the Analyzed GeneSet

  

Fig 3: GOMF\_TRANSCRIPTION\_COREGULATOR\_ACTIVITY: Random ES distribution      
 Gene set null distribution of ES for **GOMF\_TRANSCRIPTION\_COREGULATOR\_ACTIVITY**
